# Investigation of MXenes Oxidation Process during SPS Method Annealing

**DOI:** 10.3390/ma14206011

**Published:** 2021-10-12

**Authors:** Jaroslaw Wozniak, Mateusz Petrus, Tomasz Cygan, Artur Lachowski, Marek Kostecki, Agnieszka Jastrzębska, Anita Wojciechowska, Tomasz Wojciechowski, Andrzej Olszyna

**Affiliations:** 1 Faculty of Material Science and Engineering, Warsaw University of Technology, ul. Wołoska 141, 02-507 Warsaw, Poland; mateusz.petrus.dokt@pw.edu.pl (M.P.); tomasz.cygan.dokt@pw.edu.pl (T.C.); artur@unipress.waw.pl (A.L.); marek.kostecki@pw.edu.pl (M.K.); agnieszka.jastrzebska@pw.edu.pl (A.J.); anita.wojciechowska.dokt@pw.edu.pl (A.W.); andrzej.olszyna@pw.edu.pl (A.O.); 2 Faculty of Chemistry, Warsaw University of Technology, Noakowskiego 3, 00-664 Warsaw, Poland; t.wojciechowski@pw.edu.pl

**Keywords:** microstructure, oxidation, MXenes, Ti_3_C_2_

## Abstract

This paper discusses the effects of the environment and temperature of the Ti_3_C_2_ (MXene) oxidation process. The MXene powders were annealed at temperatures of 1000, 1200, 1400, 1600, and 1800 °C in argon and vacuum using a Spark Plasma Sintering (SPS) furnace. The purpose of the applied annealing method was to determine the influence of a high heating rate on the MXene degradation scheme. Additionally, to determine the thermal stability of MXene during the sintering of SiC matrix composites, SiC–C–B–Ti_3_C_2_ powder mixtures were also annealed. The process parameters were as follows: Temperatures of 1400 and 1600 °C, and pressure of 30 MPa in a vacuum. Observations of the microstructure showed that, due to annealing of the SiC–C–B–Ti_3_C_2_ powder mixtures, porous particles are formed consisting of TiC, Ti_3_C_2_^sym^, and amorphous carbon. The formation of porous particles is a transitional stage in the formation of disordered carbon structures.

## 1. Introduction

In the last decade, there has been a lot of interest in materials with a 2D structure. This is related to their unique properties and possible applications. Some of the most interesting materials with a 2D structure are the MXene phases. They were first described in 2011 [1]. They are obtained from the MAX phases, the name of which relates directly to their stoichiometry. M is a transition metal (Ti, V, Cr, Nb, etc.), A is a metal from groups 13 or 14 (Al, Si, etc.), and X is nitrogen or carbon, n = 1.2 or 3 [2,3,4]. Their structure includes M_n_ + 1X_n_ layers with strong covalent bonds. These layers are connected by much weaker M–A bonds. A significant difference in the energy of these bonds made it possible to remove A atoms, which allowed one to obtain two-dimensional crystals with M_n_ + 1X_n_ stoichiometry [5]. This MXene composition and graphene-like structure make them auspicious materials for supercapacitors [6], lithium-ion batteries [7,8,9], hydrogen storage [10], lead absorption [11], or catalysts [12].

Due to the extensive surface area of MXene, these materials are susceptible to environmental influences. Considering the significant influence of temperature on environmental factors, the thermal stability of MXene is a significant factor as it can affect the applicability of these materials. The thermal stability of MXene has been discussed in several publications. Lotfi et al. [13] simulated the oxidation of Ti_3_C_2_ MXene in various environments (dry air, wet air, hydrogen peroxide) at temperatures ranging from 1000 to 3000 K. The research showed a significant influence of the environment on the rate of the oxidation process. In the first stage of oxidation, diffusion of Ti atoms from the middle layer to the MXene surface and the formation of the Ti–O bond were observed. This resulted in the formation of carbon structures which were further degraded at higher temperatures. Naguib et al. [14] obtained similar results. The oxidation process of MXene was carried out using rapid oxidation. The process was carried out at the temperature of 1150 °C for 30 s. As a result, it was observed that anatase and disordered carbon structures are formed during oxidation. Thermogravimetric studies also confirm the MXene oxidation pattern. Li et al. [15] conducted tests of the thermal stability of MXene in air and argon at temperatures up to 1000 °C. They observed a similar oxidation pattern regardless of the atmosphere used. However, although the thermal stability at higher temperatures was greater for argon annealed samples, the authors did not observe disordered carbon structure (DCS) formation. This was explained by a much longer heating time than in the case of the flash oxidation method. Despite the influence of the atmosphere used on the thermal stability of MXene, it is generally accepted that it is stable in the range of 800–1200 °C.

A different approach to the thermal stability problem of MXene was presented by Petrus et al. [16]. They sintered SiC composites reinforced with MXene using the SPS (Spark Plasma Sintering) method. Although the composites were sintered at 1900 °C for 30 min in a vacuum, microstructure analysis revealed the presence of disordered carbon structures similar to those described by Naguib et al. [14]. The formation of carbon structures and their presence at such a high temperature are explained by the composition of the sintered SiC–C–B powder mixtures. The absence of oxide additives and sintering in a vacuum results in only a small amount of oxygen (adsorbed on the MXene surface) which favors the formation of carbon structures [17]. This can be confirmed by works describing the SPS sintering of composites on oxide matrices or with oxide additives, where MXene decomposes completely during the sintering process [18,19]. The presented works show that the thermal decomposition of MXene is relatively complicated and does not only depend on the atmosphere used or the heating method. The presence of other substances may affect the course and speed of the oxidation process.

This article aims to determine the effects of the environment and temperature on the oxidation of MXene.

## 2. Materials and Methods

### 2.1. Materials

Ti_3_C_2_-MXene powders were used to determine the effect of oxidation conditions on the rate and scheme of the oxidation process. The production scheme for Ti_3_C_2_-MXene has been described elsewhere [18]. In brief, Ti_3_C_2_-MXene preparation is carried out via acidic etching of the MAX Ti_3_AlC_2_ phase with concentrated (48%) hydrofluoric (HF) acid under a fume hood protection. The mixture is then stirred at 1000 rpm for 24 h until all available Al layers are removed. The resulting precipitate is separated from the acidic mixture and washed thoroughly with distilled water until the pH of the clay reaches about 7. Additionally, to study the degradation of MXene during the sintering process of composites on the SiC matrix, SiC–C–B–Ti_3_C_2_ powder mixtures were annealed. The powder substrate used was comprised of: Commercial β-SiC (Alfa Aesar Haverhill, Massachusetts, USA, 99.8% chemical purity, 0.42 µm average particle size), amorphous boron powder (International Enzymes Limited Hampshire, UK, 96% chemical purity, 0.39 µm average particles size), and synthetic graphite powder (Sigma Aldrich St. Louis, MO, USA, 99% chemical purity, average fake size below 20 µm).

### 2.2. Annealing Process

The MXene powder annealing process was carried out in a graphite die in an SPS furnace (HP D10, FCT System GmbH Effelder-Rauenstein, Germany) at temperatures of 1000, 1200, 1400, 1600, and 1800 °C in argon and in a vacuum. The powders were heated to the desired temperature at the rate of 100 °C/min and then, after reaching the desired temperature, quickly cooled down to room temperature. In the case of annealing of SiC–C–B–Ti_3_C_2_ powder mixtures, the following parameters of the annealing process were used: Temperatures of 1400 and 1600 °C and pressure of 30 MPa in a vacuum. After reaching the desired temperature, the powders were quickly cooled down to room temperature.

### 2.3. Powders Characterization

After annealing, Ti_3_C_2_-MXene was subjected to observation under a scanning electron microscope (SEM Hitachi 5500) (Hitachi, Tokyo, Japan). Additionally, optical absorption spectra were obtained using a UV–Vis spectrometer (Evolution 220, Thermo Scientific, Waltham, MA, USA) equipped with a Spectralon-covered ISA-220 integration sphere with a 60 mm diameter. Spectra were recorded in the range of 220–1100 nm. The measurement parameters were: Scanning speed of 200 nm min^−1^, integration time of 0.30 s, and resolution of 1.00 nm. Total diffuse reflectance spectra measurements were made, on the basis of which, the band gap was calculated using the Kubelka-Munk function. The band gap is calculated by plotting the square product of the Kubelka-Munk function and the energy F (R) hv versus the energy hv. The band gap can be obtained by extending a straight line from the straight line segment touching the x-axis [20]. The Kubelka-Munk F (R) hv function is determined from Equation (1) [21].

F(R) = k/s(1)

where k—the molar absorption coefficient k = (1 − R)^2^, R—the reflectance data from the diffuse reflectance data, S—the scattering factor, s = 2Rh, hv—energy

As with pure MXene, the SiC–C–B–Ti_3_C_2_ powder mixtures after annealing were also observed by scanning electron microscopy and analyzed using UV–Vis. Additionally, they were subjected to observations under a transmission electron microscope (TECNAI G2 F20 S-TWIN microscope operating at 200 kV) (FEI Technologies Inc., Oregon, United States).

Simulation of electron diffraction patterns and high-resolution TEM (Transmission electron microscopy) images were performed to find a possible crystal structure of the observed MXene phase. A Ti_3_C_2_ unit cell from the Material Project database [22] (material ID: mp-1094034) was chosen as a reference unit cell for the MXene phase. For electron diffraction patterns and high-resolution TEM images simulations, PTCLab software [23] and QSTEM [24] code were used, respectively. The microscope settings in the simulation inputs corresponded to the TECNAI G2 F20 S-TWIN parameters and were fixed. Modification of the atom coordinates in the reference unit cell were made to obtain the best match between the experimental data and the simulations.

## 3. Results

**Figure 1:**
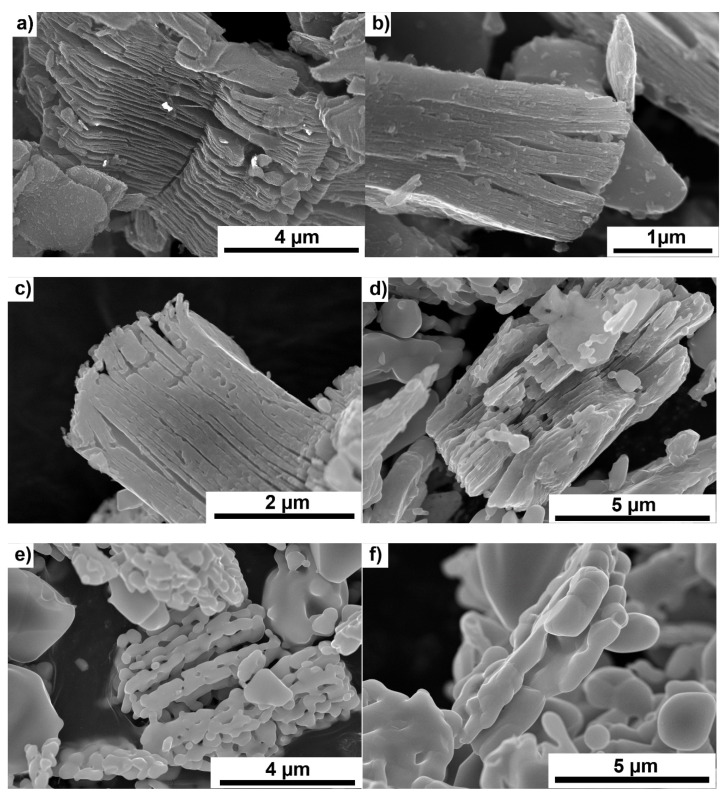
Morphology of Ti_3_C_2_ MXene powders; starting powder (**a**) powders annealed in argon at temperatures of (**b**) 1000 °C, (**c**) 1200 °C, (**d**) 1400 °C, (**e**) 1600 °C and (**f**) 1800 °C.

Figure 1a–f shows the changes of the Ti_3_C_2_-MXene powder morphology after annealing in argon. With increasing temperature, the powders gradually oxidize on the surface, resulting in a slow disappearance of the layered structure of the powders. 

**Figure 2:**
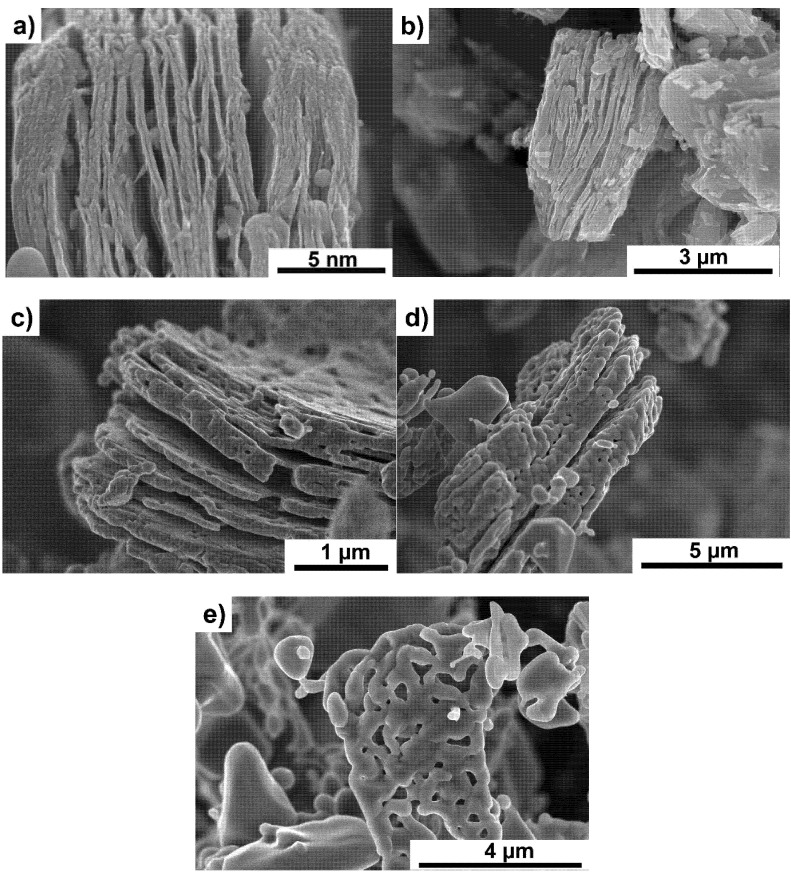
Morphology of Ti_3_C_2_ MXene powders annealed in vacuum at temperatures of (**a**) 1000 °C, (**b**) 1200 °C, (**c**) 1400 °C, (**d**) 1600 °C and (**e**) 1800 °C.

At 1600 °C, flake-shaped particles (Figure 1e) composed of smaller crystallites formed during the oxidation of MXene are visible. Powders annealed at the highest temperature (Figure 1f) lose their flake shape, which is related to the heating temperature close to the melting point of TiO_2_ [25]. A similar scheme of oxidation was observed for powders annealed in a vacuum (Figure 2a–e). Only a slightly lower degree of MXene oxidation was observed for powders annealed at the lowest temperatures of 1000 and 1200 °C. At higher temperatures, an almost identical particle decomposition was observed, as in powders heated in argon. The analysis of the obtained results showed a typical degradation of MXene. Above 1200 °C, the particles almost completely decomposed. The higher range of Ti_3_C_2_ MXene stability than reported in the literature [13] is probably related to the faster heating of the powder through the SPS method. Moreover, the occurrence of disordered carbon structures, the formation of which we described in our previous works, has not been observed.

**Figure 3:**
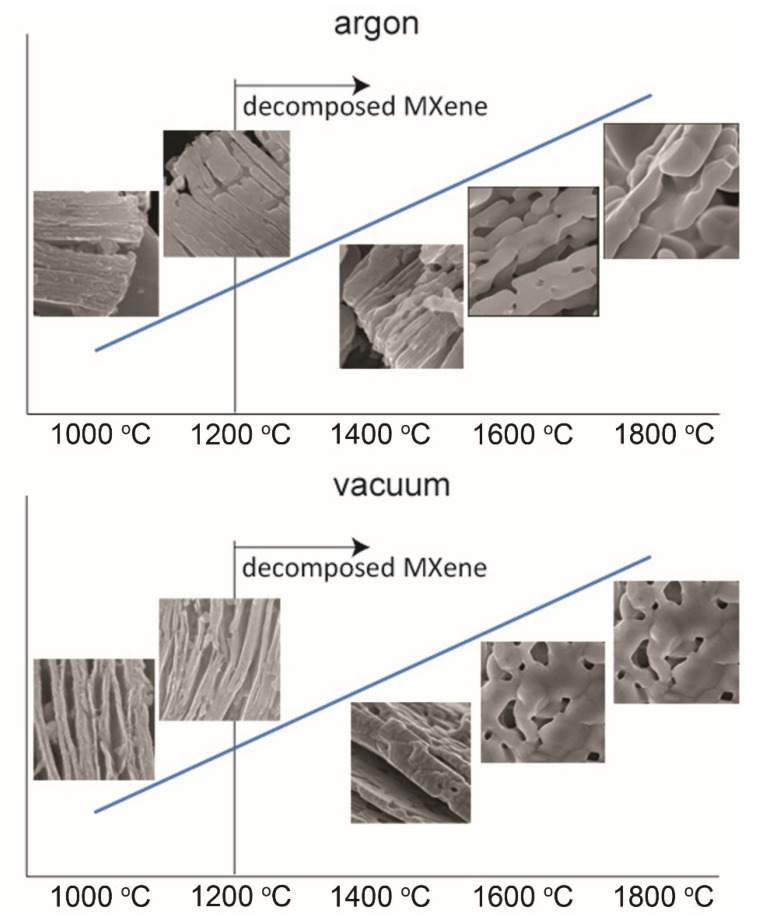
Diagram showing the decomposition steps of MXene annealed in argon and vacuum.

A comparison of the MXene degradation stages, depending on the annealing atmosphere, is presented in Figure 3. In both cases, the degradation process proceeds similarly. Above 1200 °C, MXene is completely degraded, and its layered structure will be lost. A further increase in temperature causes the formation of particles composed of equiaxed crystallites. The only differences in degradation, depending on the atmosphere, can be seen at lower annealing temperatures. In the case of a vacuum, a slightly slower degradation can be observed compared to argon-annealed powders. The differences in the scheme of MXene degradation are related to the presence of adsorbed oxygen on the MXene surface, which is responsible for the oxidation processes during annealing.

**Figure 4:**
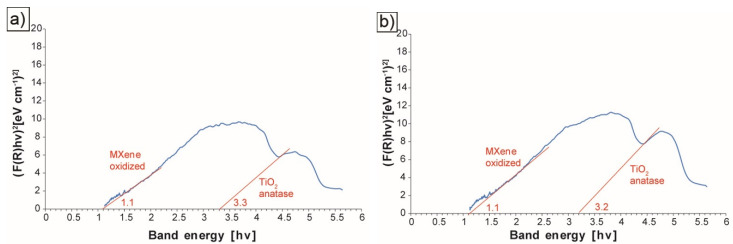
The plot of the Kubelka-Munk function in a function of band energy for (**a**) Ti_3_C_2_ MXene annealed at 1600 °C in argon, (**b**) Ti_3_C_2_ MXene annealed at 1600 °C in vacuum.

To identify the oxides present on the MXene surface, the bandgap value was determined. Exemplary test results are summarized in Figure 4a,b. By analyzing the obtained results, it can be concluded that all the tested materials contain the oxidized Ti_3_C_2_ phase. Its value for the bandgap is 1.1 for the MXene phase annealed under various conditions. In most of the tested materials, we also observe a peak for which the bandgap is from 3.2 to 3.3 related to the presence of TiO_2_ in the form of anatase on the surface of the tested materials [20]. The presence of anatase at such a high temperature (1600 °C) is quite surprising. According to the literature, the transformation of anatase to rutile takes place in the temperature range of 450–900 °C [26]. However, the transformation process is dependent on many factors, including temperature, time, heating speed, particle size, morphology, surface area, and atmosphere [27]. Considering the annealing method that ensures rapid heating to a certain temperature, the process of transforming anatase to rutile could not have taken place due to the limited time of the process.

**Figure 5:**
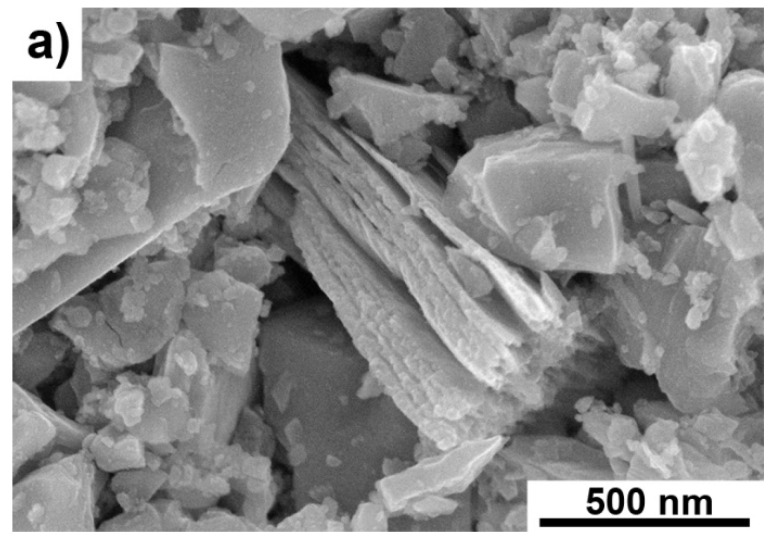

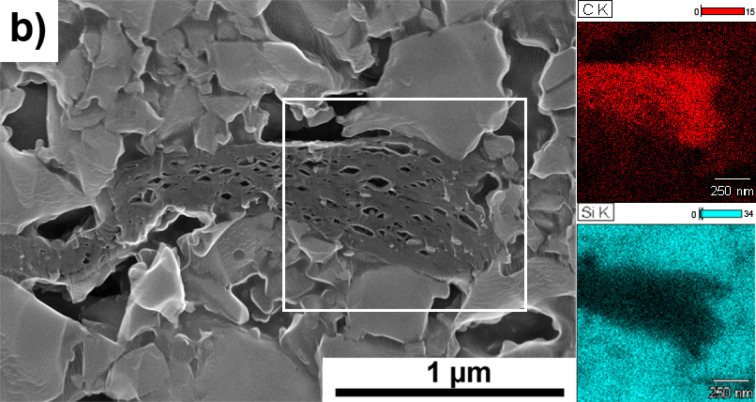
Morphology of SiC–C–B–Ti_3_C_2_ powders annealed in vacuum (**a**) at 1400 °C, (**b**) at 1600 °C.

Because the observations of the MXene powders after the annealing process did not show the presence of disordered carbon structures, similar tests were carried out for the SiC–C–B–Ti_3_C_2_ powder mixtures. This composition results from our previous work on the sintering of SiC composites reinforced with MXene, in which the presence of disordered carbon structures was observed. In these samples, due to the more complex composition (the presence of SiC, C, and B powders), the powders were observed in the form of loose powders and specially prepared samples enabling observation using the transmission electron microscope. In the case of lower annealing temperatures, the degradation of MXene occurred to a similar extent as in the case of annealing pure MXene (Figure 5a). A thicker oxide layer appears on the Ti_3_C_2_ surface. Much greater differences were observed for powders annealed at a temperature above 1600 °C. The presence of porous particles with a sponge-like structure was noted (Figure 5b). EDS (Energy-Dispersive Spectroscopy) analysis of these particles showed the presence of only carbon, which indicates a change in the degradation scheme of MXene in the presence of SiC–C–B. The presence of porous particles, with a shape similar to MXene, proves the formation of the disordered carbon structures described in our previous papers [16]. However, the morphology of these particles differs from those we observed in the SiC matrix sinters [16,17].

**Figure 6:**
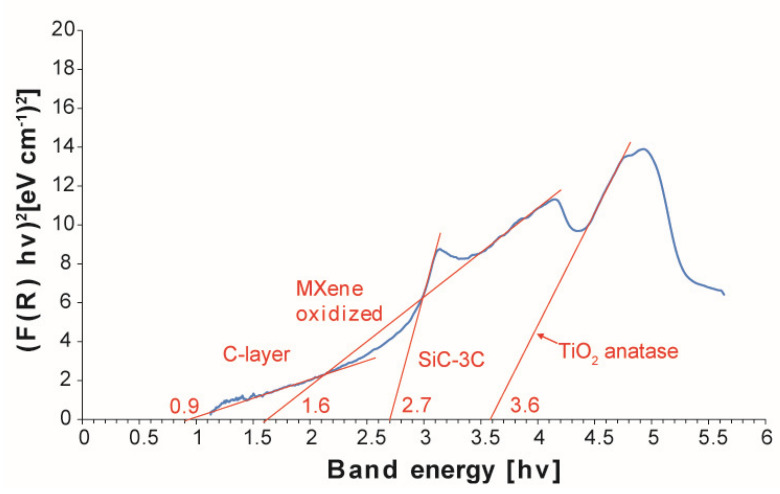
The plot of the Kubelka-Munk function in a function of band energy for SiC–C–B– Ti_3_C_2_ MXene annealed at 1600 °C in vacuum,.

The disordered carbon structure particles did not show such porosity. This may indicate that the formation of porous particles is a transitional stage in the formation of disordered carbon structures. As in the case of annealed pure MXene, the bandgap value was determined for the identification of oxides on the MXene surface (Figure 6).

Apart from oxidized MXene and anatase observed for pure powders, SiC–3C (bandgap 2.7 hv) and C-layer (bandgap 0.9 hv) were also identified. This confirms our SEM observations of the powder in which elongated particles with a high carbon content were identified. To fully characterize the observed particles, observations using a transmission electron microscope were carried out.

**Figure 7:**
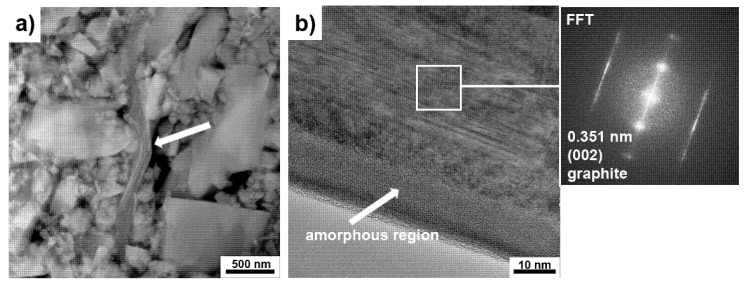
Transmission electron microscopy (TEM) analysis of SiC–C–B–Ti_3_C_2_ powder after annealing at 1600 °C: (**a**) Disordered carbon structures, (**b**) high-resolution transmission electron microscopy (HRTEM) image and fast Fourier transform (FFT) pattern of DCS.

**Figure 8:**
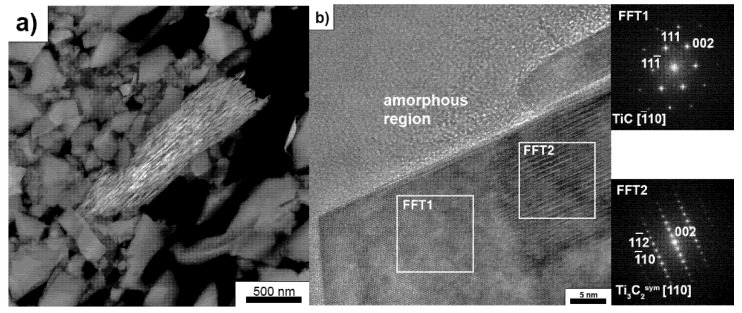
Transmission electron microscopy (TEM) analysis of SiC–C–B–Ti_3_C_2_ powder after annealing at 1600 °C: (**a**) Porous layered structures, (**b**) high-resolution transmission electron microscopy (HRTEM) image and fast Fourier transform (FFT) pattern of porous layered structures.

TEM analysis of SiC–C–B–MXene powders annealed at 1600 °C revealed the presence of two types of particles with an elongated shape. The first type of particles is presented in Figure 7a,b. TEM analysis showed that these particles are composed of carbon layers with amorphous regions on the surface of the particles. Our previous publication identified this type of particle as disordered carbon structures in the SiC matrix sinters [16]. However, it should be noted that the amount of DCS particles is much smaller compared to the second type of identified particles. The analysis of these particles is shown in Figure 8a,b. These are porous particles that have been observed with a scanning electron microscope. These particles consist of alternating elongated crystalline and amorphous regions. Analysis of the crystalline regions showed the presence of two phases.

**Figure 9:**
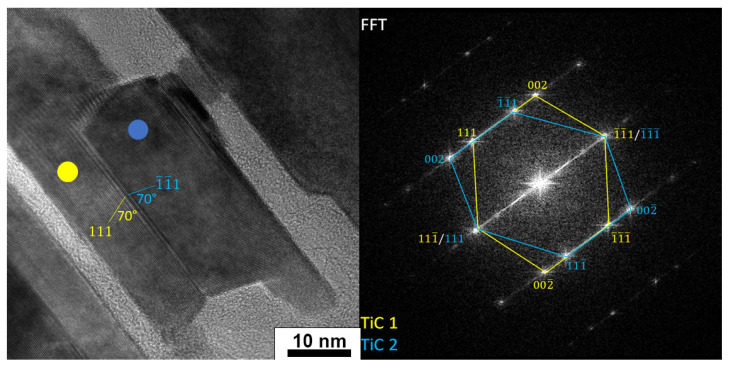
HRTEM image and fast Fourier transform (FFT) pattern of TiC twins.

The TiC was identified at the ends and in the interior areas of the elongated layers (Figure 8b) and a second phase, with a diffraction similar to that of Ti_3_C_2_. Moreover, analysis of the TiC fast Fourier transform (FFT) pattern showed the presence of a twin boundary. A similar formation of TiC twins has been described in the literature in the case of MAX phases [28]. A HRTEM image and FFT pattern of TiC twins are presented in Figure 9. The measured angles between (111) and (111) are 70°.

**Figure 10:**
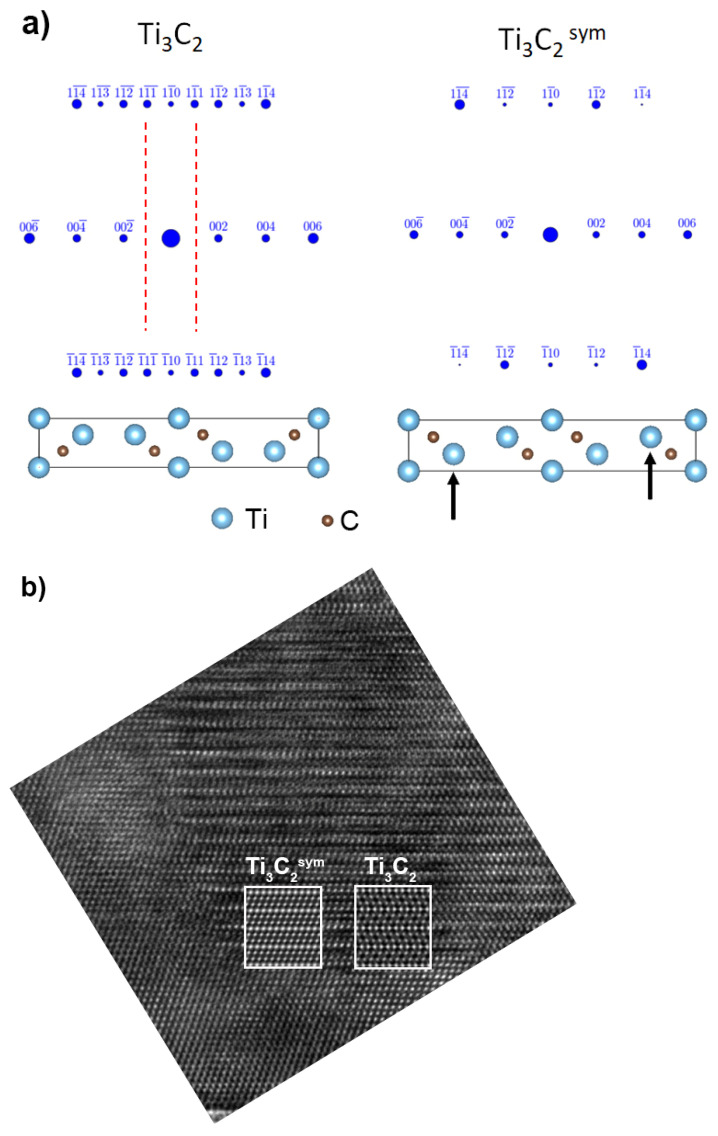
Simulation of Ti_3_C_2_^sym^ structure formation: (**a**) The way of performing simulation visualized using VESTA (ver. 3.5.7) (National Museum of Nature and Science, Tsukuba-shi, Japan) [30], (**b**) comparison of Ti_3_C_2_ and Ti_3_C_2_^sym^ structures to the structure obtained in the experiment.

However, there are some differences. As shown in the simulation for Ti_3_C_2_ (Figure 10a), there are (111) reflections in the zone axis, which were not identified on diffraction. However, it is enough to change the position of the Ti atoms (indicated by the arrows), as for Ti_3_C_2_^sym^, and the diffraction pattern will be similar to the simulation. Additionally, there is a better match of the Ti_3_C_2_^sym^ structure to the HRTEM image than Ti_3_C_2_ (Figure 10b). In the case of Ti_3_C_2_, there is a change in the orientation of the atoms not observed in the experiment, where all atoms are arranged in one direction, exactly as for the Ti_3_C_2_^sym^ simulation. Based on the obtained research results, it is possible to propose a mechanism responsible for the formation of disordered carbon structures. According to the literature data, the starting Ti_3_C_2_ powder has twin boundaries [28]. Since these are high energy boundaries, Ti atoms are shifted during the annealing process, resulting in the formation of the Ti_3_C_2_^sym^ structure, as shown in Figure 10a. Further thermal activation leads to a redistribution of carbon atoms so that there are no Ti–Ti bonds, omitting C. As a result of this process, TiC_0.67_ forms with twin boundaries and voids occur. Rapid temperature rises can occur in the voids leading to the removal of Ti, which results in the formation of amorphous carbon regions between TiC_0.67_. This process is much more efficient at the particle edges of the MXene phases. Therefore, in the internal areas, the removal efficiency of titanium is lower, and there remain areas where Ti_3_C_2_^sym^ occurs without twin boundaries. At a higher temperature, recrystallization and further removal of Ti occurs, resulting in the formation of defective carbon structures. Other authors observed a similar mechanism of decomposition as a result of the annealing of the MAX phases [28,29].

## 4. Conclusions

In this work, research was carried out to determine the mechanism of MXene decomposition during annealing. The pure Ti_3_C_2_ powders annealed in both vacuum and argon showed a similar nature of degradation. As the temperature increased, the MXene particles became covered with an increasingly thicker layer of oxides. In the case of MXene annealed in the presence of SiC, B, and C powders, the particles decompose to form porous structures composed of TiC, Ti_3_C_2_^sym^, and amorphous carbon. The formation of porous structures is a transitional stage which consequently leads to the formation of defective carbon structures. The obtained test results are consistent with those obtained in our previous studies in which we postulated the influence of SiC, B, and C on the formation of disordered carbon structures.

## Data Availability

The data presented in this study are available on request from the corresponding author.
